# Diabetes in Patients With Ataxia Telangiectasia: A National Cohort Study

**DOI:** 10.3389/fped.2020.00317

**Published:** 2020-07-09

**Authors:** Helena Donath, Ursula Hess, Matthias Kieslich, Marius Theis, Ute Ohlenschläger, Ralf Schubert, Sandra Woelke, Stefan Zielen

**Affiliations:** ^1^Division of Allergology, Pulmonology and Cystic Fibrosis, Department for Children and Adolescents, Goethe University, Frankfurt, Germany; ^2^Division of Pediatric Neurology, Department for Children and Adolescents, Goethe University, Frankfurt, Germany

**Keywords:** ataxia telangiectasia, diabetes, HbA1c, OGTT, diabetes therapy

## Abstract

**Background:** Ataxia telangiectasia (A-T) is a rare autosomal-recessive multisystem disorder characterized by pronounced cerebellar ataxia, telangiectasia, cancer predisposition and altered body composition. In addition, evidence is rising for endocrine dysfunction.

**Objectives:** To determine the evolution of diabetes and its prevalence in a larger A-T cohort.

**Methods:** A retrospective analysis of the patient charts of 39 subjects from the Frankfurt A-T cohort was performed between August 2002 and 2018 concerning HbA1c and oral glucose tolerance (OGTT). The median follow-up period was 4 years (1–16 years). In addition, in 31 A-T patients aged 1 to 38 years HbA1c and fasting glucose were studied prospectively from 2018 to 2019.

**Results:** In the retrospective analysis, we could demonstrate a longitudinal increase of HbA1c. The prospective analysis showed a significant increase of HbA1c and fasting glucose with age (*r* = 0.79, *p* ≤ 0.0001). OGTT has a good sensitivity for insulin resistance screening, whereas HbA1c can be used to evaluate individual courses and therapy response. Seven out of 39 (17.9%) patients suffered from diabetes. Metformin did not always lead to sufficient diabetes control; one patient was treated successfully with repaglinide.

**Conclusion:** Diabetes is a common finding in older A-T patients and often starts in puberty. Our data clearly demonstrate the need for an annual diabetes screening in patients > 12 years.

## Background

Ataxia telangiectasia (A-T) is a rare autosomal recessive multisystem disorder characterized by pronounced cerebellar ataxia, telangiectasia, cancer predisposition, and altered body composition (1–3). The incidence is estimated at 1:40.000–1:200.000 (4).

The sequence of the *Ataxia Telangiectasia Mutated (ATM*) gene has been known since 1995. It is located in the region q22-23 of chromosome 11 and encodes a 370 kDa serine/threonine kinase called ATM belonging to the family of signal transduction molecules (5, 6) and is activated in response to DNA double-strand breaks. ATM has over 700 interaction partners, including the tumor suppressor p53 (7, 8). In this way, a large number of processes such as cell cycle checkpoints, DNA repair systems or apoptosis are controlled. Due to the multitude of tasks, the failure of the kinase results in a complex clinical appearance that manifests in various organ systems (9). Many of the clinical alterations observed in A-T patients may be related to the dysfunctional control of reactive oxygen species (ROS) observed when ATM is deficient (10, 11).

Medical care for A-T patients has improved significantly during the last years and new treatment options rise hope to patients and physicians (4). With increasing life expectancy evolving morbidities like liver disease (12), insulin resistance (IR) (13), lipid alterations (14, 15), and cardiovascular disease (16) are coming to the fore as a typical signs of premature aging (17).

While gastrointestinal involvement, mainly dysphagia, poor weight gain, and failure to thrive have been characterized well (2, 3, 18–21), hepatic and metabolic disease is an entity taken into consideration only recently, with the clinical improvement and increased survival of A-T patients (12, 15). Recently, it was shown that ATM is also involved in metabolic and cardiovascular complications when disrupted (13, 15, 22, 23). ATM is a critical player in a multitude of cellular pathways for glucose metabolism (24–26). *In vitro* hyperglycemia led to increased activation of the ATM protein in pancreatic β-cells (27). The absence of ATM leads to dysglycemia and IR with lower Matsuda index when compared to controls while performing an oral glucose tolerance test (OGTT) (28).

ATM protein is involved in glucose transport, and lack of ATM can cause IR (24). Cytoplasmic ATM is a major upstream activator of Akt thus contributes to the translocation of cell surface glucose transporter 4 (GLUT4) to cell membrane (24). Early IR and a high prevalence of diabetes type 2 in older A-T patients as well as their family members are well known (23, 29–32). In the last years, it became evident that *ATM* gene polymorphisms are associated with higher risk of type 2 diabetes (33) and poorer response to metformin treatment (34).

The aim of this retrospective and in part prospective study was to evaluate our patient cohort for the incidence of IR and diabetes. In addition, we evaluated the therapy efficaciousness of diabetes treatment in seven patients.

## Methods

Between August 2002 and August 2018 we studied data of 39 classical A-T patients aged 1 to 38 years from the Frankfurt A-T cohort regarding HbA1c and outcome of OGTT. The parameters were taken from the available patient charts. In addition, 31 A-T patients were investigated for HbA1c and fasting glucose prospectively. The parameters were determined in the serum of whole blood.

(Pre-)Diabetes was defined according to recent International Society for Pediatric and Adolescent Diabetes (ISPAD) guidelines as pathological 2 h-postchallenge glucose, fasting glucose ≥ 126 mg/dL or HbA1c ≥ 5.7% (35).

All patients were clinically and/or genetically diagnosed with A-T according to recent World Health Organization (WHO) recommendations (36). We compared patients <12 years of age (group 1) to patients ≥ 12 years (group 2).

### Data Ascertainment

The data presented were collected from two non-interventional clinical trials at the children's hospital Frankfurt. Both trials were registered at clinicaltrials.gov 2012 (Susceptibility to infections in ataxia telangiectasia; NCT02345135) and 2017 (Susceptibility to Infections, tumor risk, and liver disease in patients with ataxia telangiectasia; NCT03357978). The studies were approved by the responsible ethics committee in Frankfurt (application number 121/12 and 504/15) and conducted following the ethical principles of the Declaration of Helsinki, regulatory requirements and the code of Good Clinical Practice.

### Statistical Analysis

For statistical analysis GraphPad Prism 5.01 (GraphPad Software, Inc.) was used. Values are presented as arithmetic means with standard deviations (SDs). For comparisons between the two study groups, two-tailed Mann-Whitney-*U* test was applied. Correlations were analyzed by Spearman's correlation coefficient. *P* ≤ 0.05 were considered significant.

## Results

### Retrospective Trial

**Figure 1:**
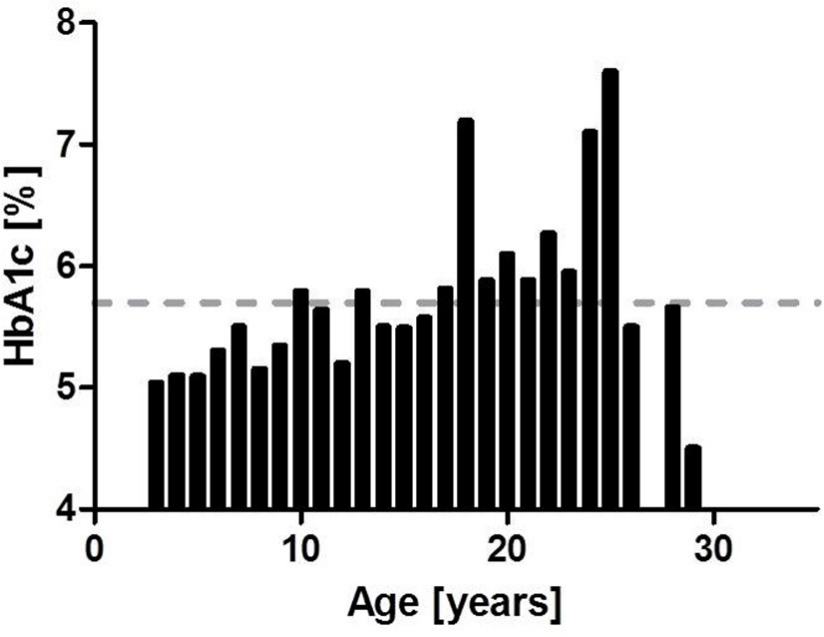
HbA1c (*n* = 3) and age. As depicted here HbA1c is increasing with age. Normal range is up to 5.7% (hatched line).

**Figure 2:**
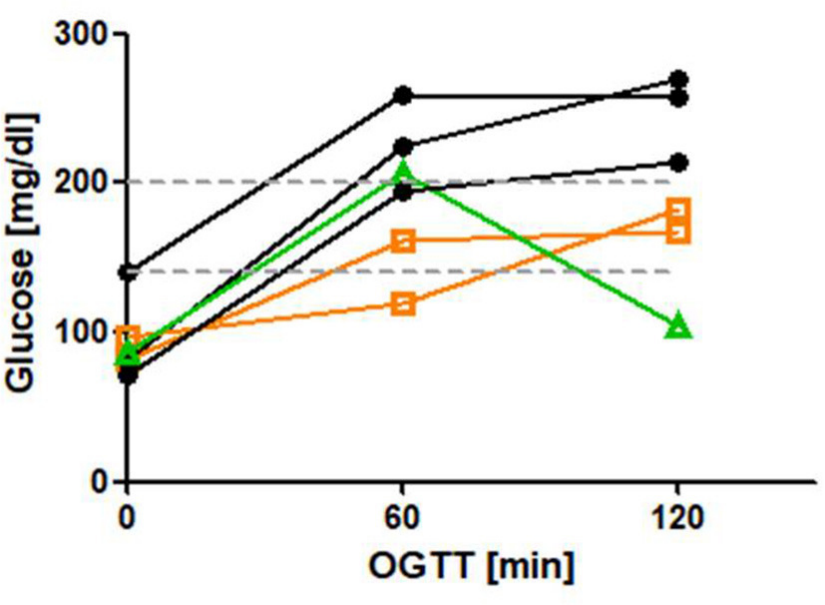
OGTT in 6 patient with diabetes. IR is defined as 120 min glucose > mg/dl, diabetes is defined as 120 min glucose > 200 mg/dl (hatched lines). *N* = *3* patients were diagnosed with diabetes (black curves). *N* = 2 had an IR and developed diabetes (orange curves). *N* = 1 had a normal OGTT and developed diabetes (green curve).

From our database, we collected HbA1c of 39 A-T patients who presented in our clinic within this period of time (2002–2018) and had at least one measurement of HbA1c; a total of 73 HbA1c measurements were performed. HbA1c was significantly higher in group 2 compared to group 1 (4.85 ± 0.14 vs. 5.65 ± 0.08%, *p* ≤ 0.0001). We could show a significant correlation between age and increased HbA1c (*r* = 0.59, *p* ≤ 0.0001). Figure 1 shows the progression of HbA1c with age. We evaluated the results of OGTT in 13 older patients (Median age: 17.5 years). As shown in Table 1, 7 out of 13 patients had IR or diabetes. OGTT was more sensitive to detect disturbed glucose metabolism than the corresponding HbA1c. All diabetic patients were ≥ 12 years of age at diagnosis (median age at diagnosis: 21 years). Figure 2 shows OGTT results in six patients who had or developed diabetes. None of the patients had auto antibodies, the family history of diabetes was unremarkable in all cases. None of the patients had systemic steroid intake documented in the patient charts.

**Table 1:** Overview about seven diabetic patient from the Frankfurt A-T cohort.

**Patient Number**	**Age at OGTT [years]**	**Result of OGTT**	**Corresponding HbA1c [%]**	**Age at diagnosis [years]**	**Metformin response**	**Cause of death**	**Clinical information**
1	17	IR	5.5	21#	Yes	-	- IR with 17 years - Metabolic syndrome with diabetes - First dietary treatment
2	19	Diabetes	5.79	19	Yes	-	- Overweight
3	18	IR	5.1	19#	Yes	-	- IR at the age of 18 - First dietary treatment
4	n.a.	n.a.	n.a.	22#	no^*^	- Lymphoma (age 26)	- First dietary treatment - Metformin non responder - Insulin glargin once daily - Repaglinide (3 x 500 mg, 30 min bevor every meal)
5	16	normal	5.56	20	Yes	-	- Overweight - Granuolma
6	21	Diabetes	6	21	no^*^	- Pneumonia with respiratory failure (age 30)	- No treatment - Recurrent pneumonia
7	25	Diabetes	4.1	25	n.a.	- Pneumonia with respiratory failure (age 25)	- No data concerning treatment and clinical follow up available

Data from the retrospective and prospective trial are shown.

* Metformin was discontinued because of gastrointestinal side effects and poor glycemic control.

^#^Diagnosed in an outpatient clinic.

*n.a., not available*.

### Prospective Trial

**Figure 3:**
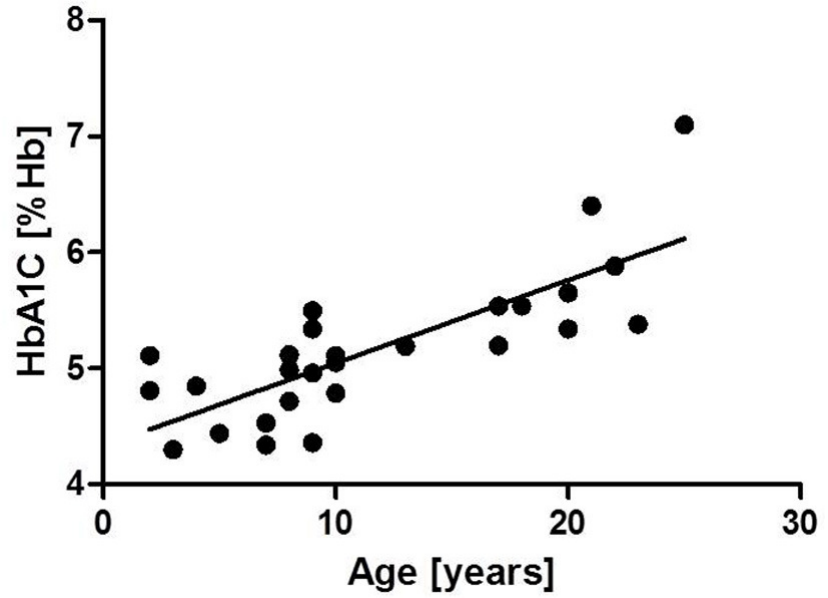
Correlation of HbA1c and age *r* = 0.79, *p* ≤ 0.0001.

**Figure 4:**
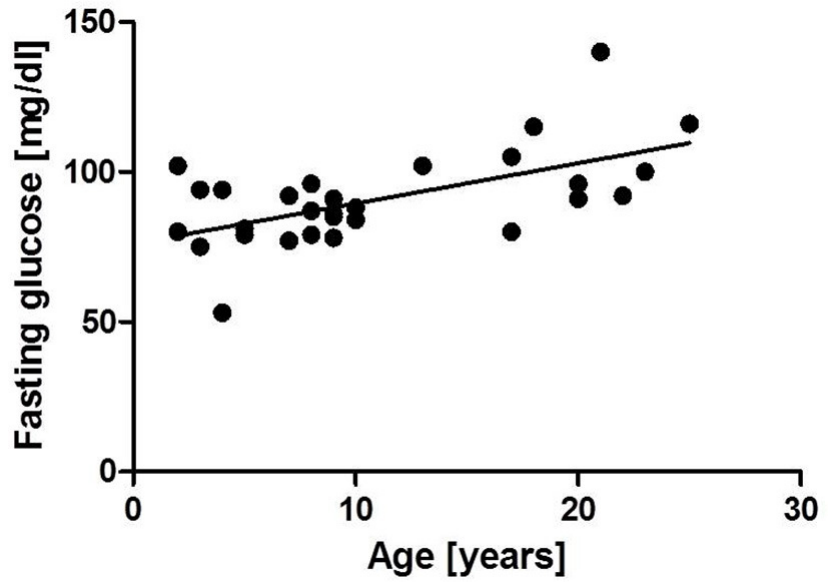
Correlation of fasting glucose and age *r* = 0.51, *p* ≤ 0.0001.

Patient characteristics are shown in Table 2. HbA1c and fasting glucose were significantly increased in group 2 compared to group 1 (HbA1c: group 1: 4.84 ± 0.35, group 2: 5.72 ± 0.6%; *p* ≤ 0.0001, fasting glucose: group 1: 84.2 ± 10.13 mg/dL, group 2: 103.7 ± 16.8 mg/dL, *p* ≤ 0.0001). Pathologically increased HbA1c levels were found in 30% (3/10) of older A-T patients. We could establish a significant correlation of HbA1c (*r* = 0.79, *p* ≤ 0.0001) and fasting glucose (*r* = 0.51, *p* ≤ 0.001) with age. The correlations are shown in Figures 3, 4. 30 % (3/10) of group 2 suffered from diabetes type 2 whereas no patient in group 1 was affected.

**Table 2:** Patient characteristics.

**Parameter**	**Age <12 years (*n* = 21)**	**Age > 12 years (*n* = 10)**	***P* value**
Sex	9♀ / 12♂	5♀/5♂	
Age [years]	6.5 ± 2.8	19.6 ± 3.5	≤ 0.0001
Weight [kg]	21.1 ± 5.0	50.4 ± 16.4	≤ 0.0001
BMI [kg/m^2^]	15.7 ± 1.5	20.3 ± 4.3	≤ 0.001
Z-Score BMI	−0.3 ± 0.8	−0.9 ± 1.2	n.s.
AFP [ng/mL] Normal range <7 ng/mL	313.4 ± 267.2	540.8 ± 275.8	≤ 0.05
HbA1c [%] Normal range <5,7%	4.84 ± 0.35	5.72 ± 0.6	≤ 0.0001
Fasting glucose [mg/dL]	84.2 ± 10.13	103.7 ± 16.8	≤ 0.0001
Diabetes Type 2	*n* = 0	*n* = 3	

Data from the prospective trial are shown.

*The values are shown as mean + SD, n.s., not significant*.

**Figure 5:**
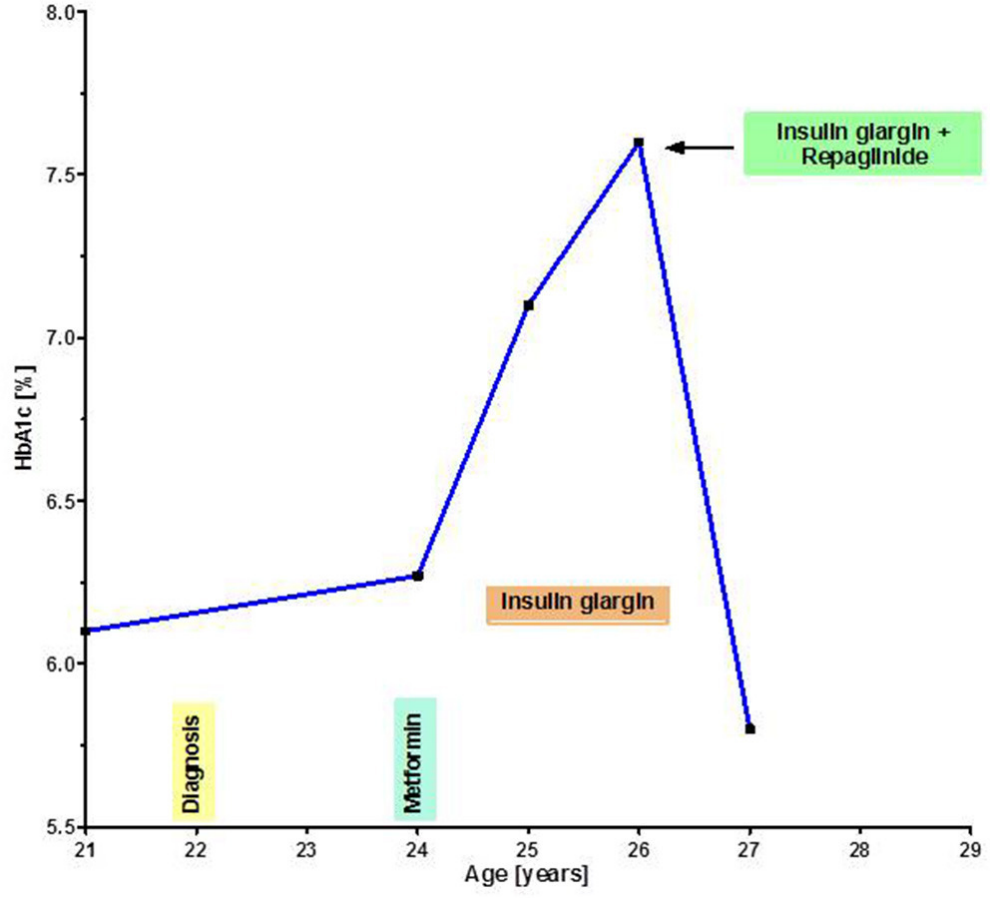
Individual course of HbA1c in a diabetic patient under different treatments. Repaglinide led to an efficacious glycaemia control.

All diabetic patients received treatment with metformin (Table 1). Response to metformin was favorable 4/7 patients (57.1%). 2/7 patients did not respond to metformin monotherapy. Due to gastrointestinal side effects, one of these patients discontinued the metformin treatment. After a short period without any treatment, subcutaneous injections of insulin glargin were started. Still, the patient suffered from poorly controlled diabetes marked by a fasting glucose of 250 mg/dL and HbA1c of 7.6% at presentation in our clinic. Due to an advanced neurological deficit we were hesitant to initiate an intensified subcutaneous insulin therapy and therefore decided to treat him with repaglinide orally. Within 8 weeks, HbA1c dropped to 6.2%. The individual course of HbA1c of this patient is shown in Figure 5.

## Discussion

A-T is a lethal, chronic degenerative disease. Due to the improved treatment options in the recent years, hitherto largely unknown disease features such as endocrine dysfunction, liver disease and cardiovascular diseases are gaining in importance (12, 15, 37, 38). The present work clearly demonstrates the high rate of type 2 diabetes (17.9%) among post-pubertal patients.

Diabetes is one of the leading causes of death worldwide (39). It leads to a high cardiovascular risk, micro-angiopathy, dyslipidemia, nephropathy, neuropathy, and repressed immune system (39). HbA1c values in the upper normal range indicate a high risk for later diabetes (40). In condition of A-T, IR and diabetes have rarely been investigated in clinical settings. In view of the comorbidities (e.g., malnutrition, neurological deficit, and immunodeficiency), consistent diabetes therapy is of particular importance.

In the cytoplasm of the cells, ATM causes activation of the serine/threonine-specific protein kinase Akt in response to insulin. Akt is an important protein which participates in the signaling cascade for the inhibition of apoptotic signals (41). In response to insulin, protein translation is stimulated, and glucose uptake is controlled by GLUT 4 (24). Mice with a muscle-specific deletion in the *GLUT 4* gene develop IR and glucose intolerance (42). Low ATM levels will therefore contribute to the development of IR and glucose intolerance in A-T via the down-regulation of Akt activity in muscle cells (24).

In 2000, the insulin signaling induced ATM-dependent phosphorylation of 4E-BP1 was reported (46). Ever since, the deficiency in the insulin and insulin-like growth factor 1 (IGF-1) axes has been demonstrated in the absence of ATM (20, 26). Apo E knockout mice without ATM protein showed increased IR and were prone to develop a metabolic syndrome (22).

Additionally, *ATM* is a regulator of adipocyte differentiation. In *Atm*-deficient mice lack of induction of C/EBPα and PPARγ, central transcription factors for adipocyte differentiation, as well a reduced fat mass were reported (43). Of course, fat mass is of particular importance for glucose metabolism and homeostasis. There was no significant difference when comparing fat mass of A-T patients to sex and age matched healthy controls in humans (2). Apparently, the significantly decreased lean mass is a major contributor to the disturbed glycemic control in A-T patients.

Apart from that, there have been few reports on endocrine abnormalities in A-T patients (38). While poor weight gain, stunting and delayed pubertal development have been characterized as a typical findings in A-T (2, 3, 19, 20, 38), abnormalities in glucose metabolism, also if known since long time, are hardly described as clinical manifestation (30–32). We have recently reported about liver involvement in A-T and dyslipidemia (12). In synopsis of lipid metabolism disorder and IR, A-T patients suffer from an incomplete metabolic syndrome with increased risk for cardiovascular events (15, 16, 44).

Due to better care, life expectancy of A-T patients has emerged over the last decades (45). Especially in the light of new treatment options such as bone marrow transplantation (46–48), dexamethasone treatment (49–51), and gene therapy (52–54) disease facets with manifestation in the later disease course should be screened and treated. According to our data, diabetes screening is indicated starting for the age of 12 years. HbA1c is an easy to obtain, inexpensive marker that can be used to evaluate individual courses and therapy response. However, OGTT is more sensitive in diagnosing IR than HbA1c and fasting glucose. This shows that the OGTT is still of value and confirms the current recommendation of the English CF society: HbA1c reflects glycemic control over a period of time. The statement about the long-term course of blood glucose has some advantages, but the values of HbA1c in CF patients may still be within the normal range when the OGTT already shows an IR or even diabetes (55). Taken these information into account, we truly believe that both measurements, HbA1c and OGTT, should be applied in A-T patients.

First line treatment for insulin-resistant diabetes is metformin (39). However, to our clinical experience, not all A-T patients respond to treatment with metformin. As has been shown in 2011, inhibition of ATM in rat hepatoma cell lines diminished the effect of metformin by reduced phosphorylation and activation of AMP-activated protein kinase (25). Additionally, the gene variant SNP rs11212617 at a locus that includes the *ATM* was proved to influence the glycemic response to metformin in type 2 diabetes (56). In line with these studies, Connelly et al. reported that the absence of ATM leads to dysglycaemia and IR with lower Matsuda index when compared to controls while performing an OGTT (28). Nevertheless, they could not show altered fasting glucose levels, insulin concentrations or insulinogenic index measurements (28).

In addition to that, it is important to consider the general condition of the patient with particular attention to the neurological status, body composition, and independence in the patients' everyday life. For instance, subcutaneous injections often present an insurmountable barrier to self-administration due neurological impairment [unpublished clinical observation]. Apart from clinical experience, research on endocrine, and metabolic alterations in A-T is rare (15, 22, 37, 57). No guidelines for treatment of diabetes in this challenging patient group are available.

In case a patient does not respond adequately to metformin therapy, insulin treatment is recommended. The beneficial effects of insulin as anabolic hormone should be taken into consideration when escalating diabetes therapy (58). Especially in malnourished patients, an amelioration of the nutritional status with weight gain could be achieved with insulin injections. The insulin/IGF-1 axis increases muscle mass and bone density and improves insulin sensitivity as well as enhancement of free fatty acid oxidations in the muscles. Also, it was shown recently that the IGF-1 pathway has beneficial effect on cardiovascular and cerebrovascular disease (59). On the other hand, insulin as anabolic hormone and growth factor may possibly increase the cancer risk in A-T patients (60).

However, self-administration of subcutaneous insulin injections are not feasible for older A-T patients with considerable neurological deficit. In case an insulin therapy is initiated, they are dependent on their caregiver. There is a dilemma between the autonomy of patients and the necessary treatment. To improve compliance, a different treatment regimen with oral antidiabetic drugs such as repaglinide may be used in special cases (61). In the Frankfurt A-T cohort, one of our A-T patients with diabetes had poorly controlled serum glucose levels under treatment with insulin glargin. We initiated a treatment with repaglinide. Hereunder, with a very favorable side-effect profile, a good therapeutic success and at the same time excellent compliance was achieved.

This study has some limitations. Due to the retrospective design, we cannot provide a complete data set for the diagnosis of type 2 diabetes, since many patients of our national cohort are admitted to our center for routine care annually or even every second year only. Still, to our best knowledge, this is the first prospective study on diabetes in 31 A-T patients and seems to confirm our retrospective analysis of longitudinal data sets of our national cohort. Due to the large number of cases, we think we have delivered reliable data that clearly demonstrate the need for an annual diabetes screening in patients ≥ 12 years.

## Conclusion

Especially with advancing age, a diabetes screening should be conducted regularly in A-T patients. IR and diabetes have to be treated in order to stabilize the nutritional status and avert further complications. OGTT has a good sensitivity for IR screening, whereas HbA1c is an inexpensive marker that can be used to evaluate individual courses and therapy response. Metformin should be administered as first line treatment and in non-responders repaglinide was shown to be safe and efficacious for glycemic control.

## At a Glance Commentary

**Scientific knowledge on the subject:** Little is known about the natural course of diabetes in ataxia telangiectasia (A-T). This is the first longitudinal retrospective and prospective surveillance of diabetes in a larger A-T cohort.

**What This Study Adds to the Field:** Diabetes is a common finding in older A-T patients and normally starts after puberty. There was a significant correlation of HbA1c and fasting glucose with age. OGTT has a good sensitivity for IR screening, whereas HbA1c can be used to evaluate individual courses and therapy response.

## Data Availability Statement

The raw data supporting the conclusions of this article will be made available by the authors, without undue reservation.

## Ethics Statement

The studies involving human participants were reviewed and approved by Ethikkommitee des Universitätsklinikums Frankfurt. Written informed consent to participate in this study was provided by the participants' legal guardian/next of kin. Written informed consent was obtained from the individual(s) for the publication of any potentially identifiable images or data included in this article.

## Author's Note

This manuscript has been released as a pre-print at Research Square, Donath et al. (62).

## Author Contributions

HD, SW, UH, SZ, and RS did the study design, data collection and interpreted, and did statistical analysis. MT, MK, UO, HD, SW, UH, and SZ conducted visits. HD and SZ wrote the manuscript. All authors read and approved the final manuscript.

## Conflict of Interest

The authors declare that the research was conducted in the absence of any commercial or financial relationships that could be construed as a potential conflict of interest.

## Abbreviations

A-T: Ataxia telangiectasia
ATM: Ataxia telangiectasia mutated
ROS: Reactive oxygen species
IR: Insulin resistance
OGTT: Oral glucose tolerance test
GLUT4: Cell surface glucose transporter 4
ISPAD: International Society For Pediatric And Adolescent Diabetes
WHO: World Health Organization
SD: Standard deviation
IGF-1: Insulin-Like Growth Factor 1
SNP: Single nucleotide polymorphism.
